# Motivational determinants of physical education grades and the intention to practice sport in the future

**DOI:** 10.1371/journal.pone.0217218

**Published:** 2019-05-23

**Authors:** Luis Cid, Ana Pires, Carla Borrego, Pedro Duarte-Mendes, Diogo S. Teixeira, João M. Moutão, Diogo Monteiro

**Affiliations:** 1 Sport Sciences School of Rio Maior, Polytechnic Institute of Santarém (ESDRM-IPSantarém), Rio Maior, Portugal; 2 Research Centre in Sports Sciences, Health and Human Development (CIDESD), Vila Real, Portugal; 3 Schools Group of D. António Ataíde of Castanheira Ribatejo, Vila Franca Xira, Portugal; 4 Life Quality Research Centre (CIEQV), Santarém, Portugal; 5 Department of Sport and Well Being, Polytechnic Institute of Castelo Branco (ESE-IPCB), Castelo Branco, Portugal; 6 Sport, Health & Exercise Research Unit (SHERU), Polytechnic Institute of Castelo Branco, Castelo Branco, Portugal; 7 Faculty of Physical Education and Sport, Lusófona University (ULHT), Lisboa, Portugal; University of Kentucky, UNITED STATES

## Abstract

Self-Determination Theory (SDT) is amongst motivational frameworks the most popular and contemporary approach to human motivation, being applied in the last decades in several domains, including sport, exercise and physical education (PE). Additionally, Achievement Goal Theory (AGT) has presented evidence of how contextual factors may influence student’s behavior in this particular context. The main purpose of this study was to analyze the motivational climate created by the teacher in the classroom, students’ satisfaction of Basic Psychological Needs (BPN), and how their behavioral regulation could explain PE grades and intention to practice sports in the future. Method: A total of 618 students (290 female; 328 male) from the 6^th^ to the 9^th^ school level, aged between 10 and 18 years (*M* = 13.3; *SD* = 1.7) participated in this study. The following surveys were used for the proposed variables: Learning and Performance Orientations in Physical Education Classes Questionnaire (LAPOPECQ); the Basic Psychological Needs in Exercise Scale (BPNESp); and the Perceived Locus of Causality (PLOCp). Intentions to practice sport/physical activity in the future were assessed through a single item. Students’ PE grade was obtained through his/her teacher final assessment. Structural Equation Analysis was performed via AMOS 23.0. Results: After analyzing modification indices and model adjustment, the final model emerged: learning climate > BPN > autonomous motivation > intentions/PE grade. Results interpretation seems to indicate that i) the satisfaction of BPN are influenced by motivational climate (i.e., learning climate), ii) the individuals’ motivation is influenced by the satisfaction of three basic psychological needs (i.e., particularly competence), and iii) the motivational regulations have direct and significant effects with intention to practice sports outside school in the future and PE grades. Discussion & Conclusion: The main results showed that a climate oriented for learning has a positive impact on basic psychological needs satisfaction of students. However, only competence satisfaction had a significant positive relationship with students’ autonomous motivation, which in turn had a significant positive relation with PE grade, as well as for intentions for leisure-time sport/physical activity practice.

## Introduction

According to several authors, Self-Determination Theory (SDT) [1] and Achievement Goal Theory (AGT) [2] are the most popular and contemporary theoretical approaches used to examine motivational processes, particularly in the physical education (PE) context [3,4,5]. Looking at physiological processes, it is through PE that most children experience a wide range of motor skills. Although its contribution is essential for the child’s development, it is not entirely clear how PE influence students’ academic performance, as well as in leisure-time physical activity. On one side of the coin, denying students’ choice for other activities due to rigid application of curricular programs may create some barriers that has some influence in the development of more self-determined or autonomous forms of motivation [6,7]. On the other side, understanding the links between social factors such as classrooms’ motivational climate encouraged by teachers, and students’ behavioural regulation, seems to be essential, since studies suggest that a targeted climate for learning (also known as mastery or task), forecasts students self-determined motivation and can have positive consequences on the practice of physical activity in general [8,9].

### Self-Determination Theory (SDT)

SDT, developed by Deci and Ryan [1], is a macro theory about human motivation which has been applied in recent years in several domains of physical activity, including PE. According to their authors [10,11,12], individuals’ motivation is not directly related to social factors (e.g., motivational climate), but are mediated by the satisfaction of “fundamental nutriments” [12]: the basic psychological needs for autonomy (i.e., capacity to regulate their own actions), competence (i.e., capacity of effectiveness in the interaction with the involvement) and relatedness (i.e., capacity of searching and developing connection and interpersonal relationships). These needs are assumed to determine differentiated behavioural regulations of an individual, encompassed in a motivational continuum that varies among several types of motivation: amotivation, external, introjected, identified, integrated, and intrinsic motivation.

According to Deci and Ryan [13], within SDT motivation can be distinguished between autonomous motivation (intrinsic, integrated and identified motivation) and controlled motivation (introjected and external motivation). In the first case, the individual manages his behaviour by self-decision and will, but in the second case, the individual feels pressured to act in an external or self-imposed way.

SDT grounded as a meta-theory emphasizes the importance on how human beings use their own resources for behavioural self-regulation, which involves the satisfaction of three basic needs, namely, autonomy, competence, and relatedness. These “nutriments” are the basis of autonomous motivation and apparently are essential for personal growth, optimal functioning, and integration of behaviour. For Ryan and Deci [12], intrinsic motivation is the most important factor on behavioural maintenance over time. Furthermore, people who regulate their motivation autonomously show more persistence, commitment, effort and pleasure in the activities they perform [14].

### Achievement Goal Theory (AGT)

Developed by Nicholls [2] and applied in sport by Duda and Nicholls [15], Achievement Goal Theory has its basis on the existence of two types for achieving goals, reflecting the criteria by which individuals assess their competence and define success or failure of their participation in a specific domain (e.g., in PE classes). The subjective judgment of achievement is of utmost importance to the individuals’ involvement in a specific activity, since it influences their motivation and has a significant impact on their behaviour [5].

At the dispositional level (motivational orientation), we can say that individuals who are task oriented (learning) focus their behaviour in improving their personal skills, and their perception of competence derives from their commitment, effort and persistence. The individuals that are ego oriented (performance) focus their behaviour in a result that comes from their involvement in the activity, and their perception of competence derives from the comparison with others. According to Duda [16, 17], results arising from the application of this theory can help predict positive consequences (or potentially negative) on behaviour, health, and well-being associated with participation in physical activity.

At the contextual level (focused on the present study), we can define the concept of motivational climate as the psychological environment induced by significant others in a specific context, that directs individual to act on a given orientation (task/learning or ego/performance). According to Standage et al. [18] and Duda [17], in a climate where emphasis is put on effort, improvement, cooperation and self-referenced goals, there is a development oriented for the task, and consequently, the individual tend to adopt adaptive strategies (more effort on actions, choose challenging tasks, more persistence on the behaviour, and better performance). On the other hand, in a climate where emphasis is placed on social comparison and results, individuals are susceptible to endorse in maladaptive strategies of achievement (less persistent, less commitment, increased anxiety, and worse performance).

### Integration of SDT and AGT in the physical activity context

According to Kingston et al. [3] and Almargo et al. [4], results from the integration of both theories in physical activity context revealed that task orientation shows a higher correlation with autonomous regulations, and individuals’ ego oriented, although with less conclusive results, show a higher correlation with more controlled forms of motivation.

Over the years, the research conducted in different populations, including exercisers [19], college athletes [20], secondary school students [21] or elementary school students [22,23], have demonstrated that either dispositional or situational achievement goals are associated with different levels of self-determination. Thus, since BPN influence behavioural regulations, one could speculate that achievement climates could have a significant influence on basic needs likewise. Looking for answers, several authors [20,24,25,26,27,28] have integrated both theoretical models to identify the associations between variables that underlie their framework on several outcomes, including the individual’s performance and intentions for future practice.

In order to analyse the predictive value of SDT and AGT variables, Biddle et al. [24] conducted a study with 723 students from Hungary, concluding that the most autonomous forms of motivation are those that best predict intentions to practice physical activity. In addition, task orientation, through identified and intrinsic regulation, show strong correlation with intention to practice. In their opinion, identified regulation is a key aspect when it comes to free choice of achievement in PE and sports context.

The basic psychological needs is a mediator between social factors (cooperative learning vs. focused on improving results) and behaviour regulation (type of motivation), as well as behavioural consequences (stress, boredom and intention to be physically active in adult life) was assessed in a study conducted by Ntoumanis [20]. In this study, which was attended by 424 students from England, the main results led the author to conclude that positive social factors (cooperative learning) result in higher grade in PE classes. Moreover, the perception of competence had a key role in PE and the need to be competent predicted autonomous behaviour. Additionally, when students experience intrinsic motivation in PE classes, positive results emerge, like more intentions of being physically active in adulthood. On the other hand, students who express more controlled motivation for PE classes have lower perception of competence, resulting in higher probability to endorse in sedentary lifestyles on the long-run.

Ntoumanis [25] conducted another study with 460 students from England, aiming to analyse the influence of motivational variables (personal and contextual) in behavioural experiences in PE classes, as well as participation in leisure-time sport activities. The author concluded that the support of the autonomy given by PE teachers, the satisfaction of basic psychological needs and the autonomous motivation, promotes positive behavioural results in mandatory PE classes. Furthermore, autonomous levels significantly predicted intentions to practice leisure-time sports activities.

In a study carried out by Fernandes et al. [26], with 1099 students of basic and higher education, the authors sought to establish the importance of perception of competence and autonomous motivation, in order to better understand the factors that determine intentions for leisure-time sport practice. Some of the main results showed that both task orientation and autonomous motivation provided the development of intentions to participate in future sport activities.

In overall, according to Ntoumanis [20] and Duda [17], studying the criterion of achievement and perception of success on different types of self-determination can create evidence to support the integration efforts of these two theories. However, there are still plenty of gaps in this research area, particularly in the PE context. Maybe this was why Standage et al. [18] stated that the perception of the motivational climate may have an important role determining motivation status, and future research should examine this in detail. According to them and more recently to Baena-Extremera, Gómes-Lópes, Graneo-Gallegos and Martínez-Molina [29], and Serrano et al. [23], the evidence points to a strong relationship between task involvement and the most self-determined form of motivation in the PE context.

### Present study

**Fig 1:**
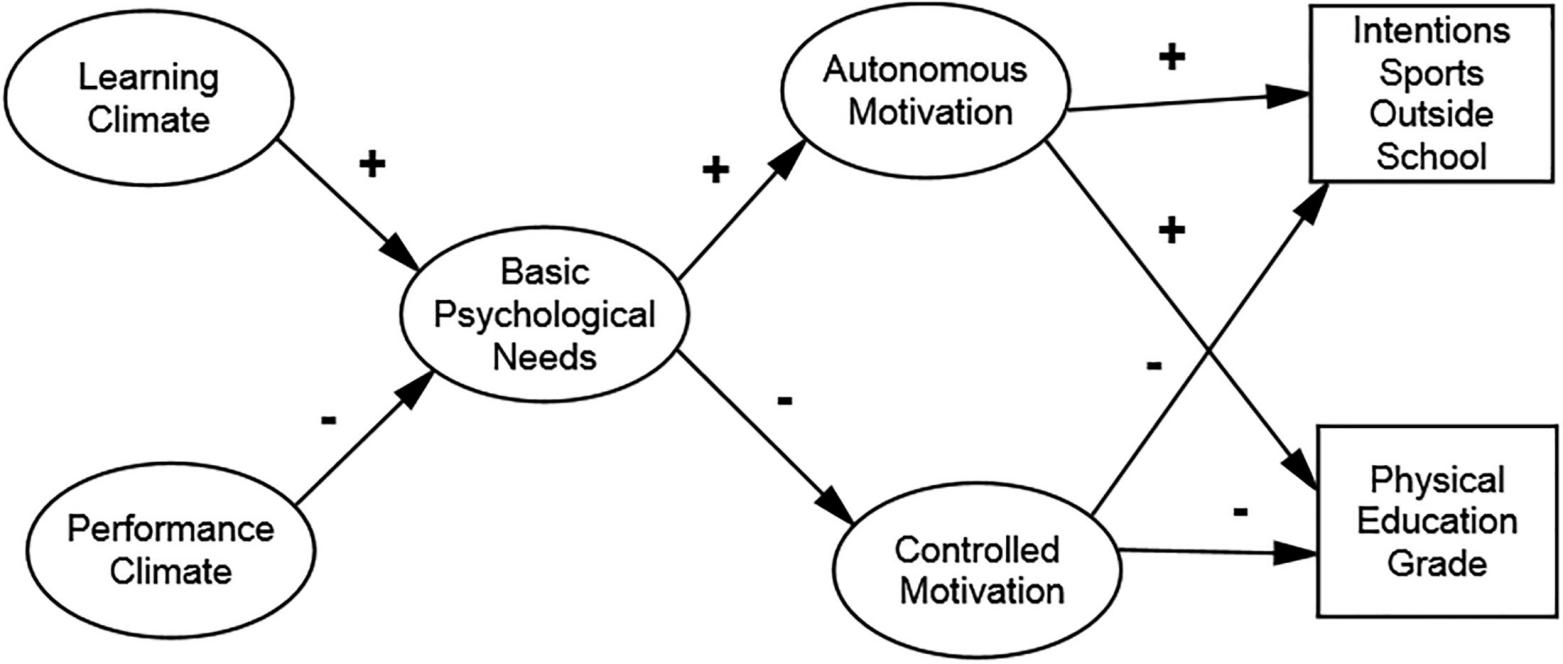
Hypothesized model.

Grounded on AGT (learning and performance climate) and SDT (basic psychological needs satisfaction and different types of motivation) principles, the aim of the present study was to understand motivational determinants of intentions to practice sports outside of school in the future and the PE grade. We also propose to analyse the invariance between gender to determine the stability of the model in both male and female students. More specifically, we propose the following hypotheses: 1) learning climate and performance climate should be positively and negatively associated respectively to the basic psychological needs satisfaction [17,18,20,30,31]; 2) basic psychological needs satisfactions should be positively associated with autonomous motivation and negatively with controlled motivation [1,10,12]; 3) in turn autonomous motivation should be associated positively with intention to practice sport in the future [11,25,27, 28,30] and PE grade [11], however controlled motivation should be associated negatively with intention to practice sports in the future and PE grade [11,28]. Also, based on both theoretical frameworks’ principles as well as on some empirical studies [28], these associations should hold true between male and female students. Fig 1 represents the proposed theoretical model under analysis to verify the aforementioned associations.

## Method

### Participants

A total of 618 students (328 female; 290 male) aged between 10 and 18 years old (*M* = 13.33; *SD* = 1.69), from Portuguese public schools (6^th^ to 9^th^ year level), participated in this study (see relevant sample characteristics presented in Table 1).

**Table 1:** Relevant sample characteristics.

N	Ages	Gender		School Level	School Extracurricular Sport Activities	Sports Practiced outside of School
		male	female			
618	10–18 (*M* = 13.33;*SD* = 1.69)	290	328	6^th^ (n = 213) 7^th^ (n = 139) 8^th^ (n = 159) 9^th^ (n = 107)	96	310

Note. N = sample size; M = mean; SD = standard deviation

Besides the fact that all of the students assiduously attended PE classes, 96 students participated in school extracurricular sports activities: handball (*n* = 15); basketball (*n* = 16); volleyball (*n* = 24); soccer (*n* = 19); dance (*n* = 12); skating (*n* = 10). Their sport experience ranged from 1–60 months (*M* = 18.11; *SD* = 1.31), weekly training ranged from 1–3 sessions (*M* = 1.62; *SD* = 0.72), and volume training varied from 30–120 minutes per session (*M* = 65.83; *SD* = 28.61).

Furthermore, 310 students stated that they also participated in sports outside of school: soccer (*n* = 69); swimming (*n* = 108); basketball (*n* = 16); combat sports (*n* = 18); gymnastics (*n* = 24); dance/ballet (*n* = 26); equestrian (*n* = 18); volleyball (*n* = 16); badminton (*n* = 15). Their sport experience ranged from 1–120 months (*M* = 41.43; *SD* = 31.83), weekly training ranged from 1–6 sessions (*M* = 2.71; *SD* = 1.21), and volume training varied from 30–150 minutes per session (*M* = 80.22; *SD* = 26.22).

### Instruments

Students’ perceptions about Motivational Climate. The Perception Learning and Performance Orientations in Physical Education Classes Questionnaire (LAPOPECQ)[8,32] Portuguese version [33] was used. This questionnaire is composed by 12 items, which are answered in a five point Likert scale, ranging from 1 (*"I totally disagree"*) to 5 (*"I totally agree"*). The items are grouped in two factors (six items each): learning climate (based on self-referenced criteria) and performance climate (based on normative criteria), reflecting the two distinct forms of student´s perception about motivational climate induced by teacher, according to AGT. In the present study, the measurement model showed the following fit adjustment to the data: χ^2^ = 70.14; df = 34; SRMR = .040; NNFI = .957; CFI = .967; RMSEA = .042; RMSEA CI 90%: .028-.055; and values of composite reliability (CR) showed acceptable internal consistency: Learning Climate = .67 and Performance Climate = .76.

Student’s Basic Psychological Needs Satisfaction. The Basic Psychological Needs in Exercise Scale (BPNES) [34], translated and validated in Portuguese by Moutão et al. [35] was used. However, for the present research items were adapted to PE context [36,37], keeping the original 12 item structure. Items are answered on a five-point Likert scale varying from 1 *(“I totally disagree”)* to 5 *(“I totally agree”)*, and grouped in 3 factors (4 items each), reflecting BPN based on SDT. In the present study, the measurement model showed the following fit adjustment to the data: χ^2^ = 204.41; df = 51; SRMR = .062; NNFI = .925; CFI = .942; RMSEA = .070; RMSEA CI 90%: .060-.080; and values of CR showed acceptable internal consistency: Autonomy = .69, Competence = .77; Relatedness = .88.

Student’s Motivational Regulation. The Perceived Locus of Causality [38] Portuguese version [6] was used, composed by 20 items which were answered on a Likert scale varying from 1 (*“I totally disagree”*) and 7 (*“I totally agree”*). The items were grouped in 5 factors (4 items each), assessing behavioral regulations based on the motivational *continuum* of SDT. In the present study, the measurement model showed the following fit adjustment to data: χ^2^ = 527.14; df = 160; SRMR = .072; NNFI = .900; CFI = .904; RMSEA = .061; RMSEA CI 90% (.055-.067); and values of CR showed acceptable internal consistency: amotivation = .82, external regulation = .72, introjected regulation = .70, identified regulation = .79, intrinsic motivation = .69.

Student´s intentions. To evaluate future intentions for the practicing sports out of school in the future, one item was developed to which students answered on a Likert scale that varied from 1 *(“No*, *certainly not”)* and 7 *(“Yes*, *absolutely certain”)*: *“It is my intention to practice (or continue to practice) sports out of school (in a club or association)*, *during the following months*, *at least 1 to 2 times per week”*. This item was formulated according to Ajzen’s [39] recommendations for creating items on assessing intention. Past studies in the PE context, likewise used one item to evaluate students’ behavioral intentions [20,24,25].

Student’s PE grade. This grade was obtained through his/her teacher final assessment, that reflect evaluation at PE during the whole year, ranging between “1” (lower grade) and “5” (higher grade). At the end of the year, students are approved to the PE class if they have a grade greater than or equal to “3”.

### Procedures

#### Data Collection

Data were collected in several schools in the two largest cities of the region where the study was conducted: Northern region of Lisbon (Vila Franca Xira) and Western region (Caldas da Rainha). All participants were recruited by convenience at PE classes.

After explaining the study’s objectives and receiving authorization of the Schools’ Executive Councils, all parents or student guardians were contacted by the respective class directors. Written consent was obtained, authorizing their children/students to participate in this research, since almost of the participants were underage. To increase reliability in the answers given and to guarantee data confidentiality, information was collected anonymously.

The assessment instruments were applied by the researchers and research assistants always in places and conditions similar to those of all participants (i.e., always in classrooms and with a maximum of 30 students), where the appropriate conditions were ensured so that the individuals did not feel strangers to the situation and, at the same time, could be concentrated during the completion of the questionnaires, which took on average about 20 minutes to complete.

Ethical approval from the committee of the Research Center in Sports Sciences, Health Sciences and Human Development (CIDESD) was obtained, under the reference UID/DTP/04045/2013.

#### Statistical analysis

Descriptive statistics (means and standard deviations) and correlations were performed for all of the variables under analysis. A Confirmatory Factor Analysis (CFA) and a Structural Equation Model (SEM) using the maximum likelihood (ML) method were performed. The recommendations of several authors [40,41,42] were followed: chi-squared test (χ^2^), degrees of freedom (df), level of significance (p), and also the following goodness-of-fit indices: Standardized Root Mean Square Residual (SRMR), Comparative Fit Index (CFI), Non-Normed Fit Index (NNFI), Root Mean Square Error of Approximation (RMSEA), and its respective confidence interval (90% CI). In the current study, we used the subsequent cut-off values as suggested [40,41,42]: SRMR ≤ .08, CFI and NNFI ≥ .90, and RMSEA ≤ .08. The analyses were conducted using SPSS 23.0 and AMOS 23.0.

Multi-group analysis was also performed to demonstrate that the re-specified models could be replicated in different groups, as suggested. For the multi-group analysis across gender, the structural invariance procedure suggested by Byrne [40] was used using the following criterion: (1) the model should fit in each sample according to adjustment indices; and (2) the differences between the unconstrained model and the models with constraints (measurement weights; structural weights; measurement intercepts; structural residuals and measurement residuals) should be ΔCFI ≤ .01, as suggested by Cheung and Rensvold [43].

## Results

### Preliminary analysis

A preliminary inspection of the data revealed that missing values comprised 0.1% of cells in the original data, without any missing data patterns. Consequently, missing data were imputed using AMOS 23.0 regression procedure. Item-level descriptive statistics indicated no deviations from univariate normality for all samples under analysis (skewness values ranged from -2 to +2; kurtosis values ranged from -7 to +7) [41]. However, Mardia’s coefficient for multivariate kurtosis exceeded expected values for multivariate normality assumptions (>5) in all samples [40]. Therefore, Bollen-Stine bootstrap of 2000 samples was employed in the subsequent analysis [44]. In addition, variance inflation factors were assessed to verify possible collinearity issues within study variables, where in this case, scores were below 1.13, showing acceptable conditions to conduct regression analysis (variance inflation factors < 10) [41]. Additionally, to determine the required sample size a G*Power analysis was performed [45] and the following parameters were considered: effect size f^2^ = 0.1; α = .05; statistical power = .95 and five predictors. Therefore, the minimum required sample should be 204, which was respected in present study.

### Descriptive and correlational analysis

According to Table 2, mean values indicate that students perceive more of a learning (*M* = 4.26; *SD* = 0.52) compared to a performance (*M* = 2.36; *SD* = 0.82) motivational climate. Results also show high means of BPN satisfaction (*M* = 3.72; *SD* = 0.53), as well as high levels of autonomous motivation (*M* = 5.53; *SD* = 0.99) compared with controlled motivation (*M* = 4.04; *SD* = 1.24), although we may consider these values as moderate.

**Table 2:** Mean, standard deviations, and bivariate correlations between the study variables.

	M±SD	MCL	MCP	BPN	AM	CM
Motivational Climate—Learning (MCL)	4.26±0.52	-				
Motivational Climate—Performance (MCP)	2.36±0.82	-.26**	-			
Basic Psychological Needs (BPN)	3.72±0.53	.39**	.04	-		
Autonomous motivation (AM)	5.53±0.99	.47**	-.06	.49**	-	
Controlled motivation (CM)	4.04±1.24	.07	.39**	.16**	.17**	-
Intentions to practice Sport	5.37±1.89	.17**	-.03	.24**	.29**	.06
Physical Education Grade	3.49±0.64	.19**	-.19**	.30**	.21**	-.21**

Note.

**p < .01

Looking at correlations (Table 2), positive and significant correlation between learning oriented motivational climate and BPN, (r = .39), as well as the autonomous motivation (r = .47), and also with intentions for practice sport in the future (r = .17) and PE grade (r = .19) were found. Contrarily, performance oriented motivational climate was positively correlated with controlled motivation (r = .39) and negatively with students’ PE grades (r = -.19). It is worthy to mention that BPN and autonomous motivation are positively correlated with intentions to practice sports and PE grades.

### Multivariate analysis

Table 3 displays the fit adjustment indices, were the initially hypothesised structural model did not fit the data. Therefore, considering that one of the objectives of using SEM is to provide additional answers beyond the validity of the models [41], residual values and the modification indices were analysed. The re-specified model (i.e., model 3) provided a good fit to the data for all samples under analysis (see Table 3).

**Table 3:** Model fit indices for the hypothesized models.

	χ^2^	df	B-S p	χ^2^/df	SRMR	NNFI	CFI	RMSEA	90% IC
Model 1	1293.3	427	< .001	3.02	.10	.80	.82	.057	.054-.061
Model 2*	330.8	117	< .001	2.82	.05	.89	.90	.054	.047-.061
Model 3**	659.6	271	< .001	2.43	.07	.90	.91	.048	.044-.053
Model 2* (FS)	244.6	117	< .001	2.09	.05	.90	.92	.058	.048-.068
Model 2* (MS)	261.3	117	< .001	2.23	.05	.90	.90	.065	.055-.076
Model 3** (FS)	514.9	271	< .001	1.90	.07	.90	.91	.055	.049-.062
Model 3** (MS)	598.6	271	< .001	2.20	.07	.90	.91	.065	.058-.072

Note.

* Without item 11 of autonomous motivation.

** Without item 11 of autonomous motivation and also item 9 of basic psychological need of autonomy; FS = female sample; MS = male sample; χ^2^ = qui-quare; df = degrees of freedom; B-Sp = bootstrap Bollen-Stine (2000 samples); χ^2^/df = normalized chi-square; SRMR = Standardized Root Mean Squared Residual; NNFI = Non-Normalized Fit Index; CFI = Comparative Fit Index; RMSEA = Root Mean Squared Error of Approximation; 90% IC = Interval Confidence.

**Fig 2:**
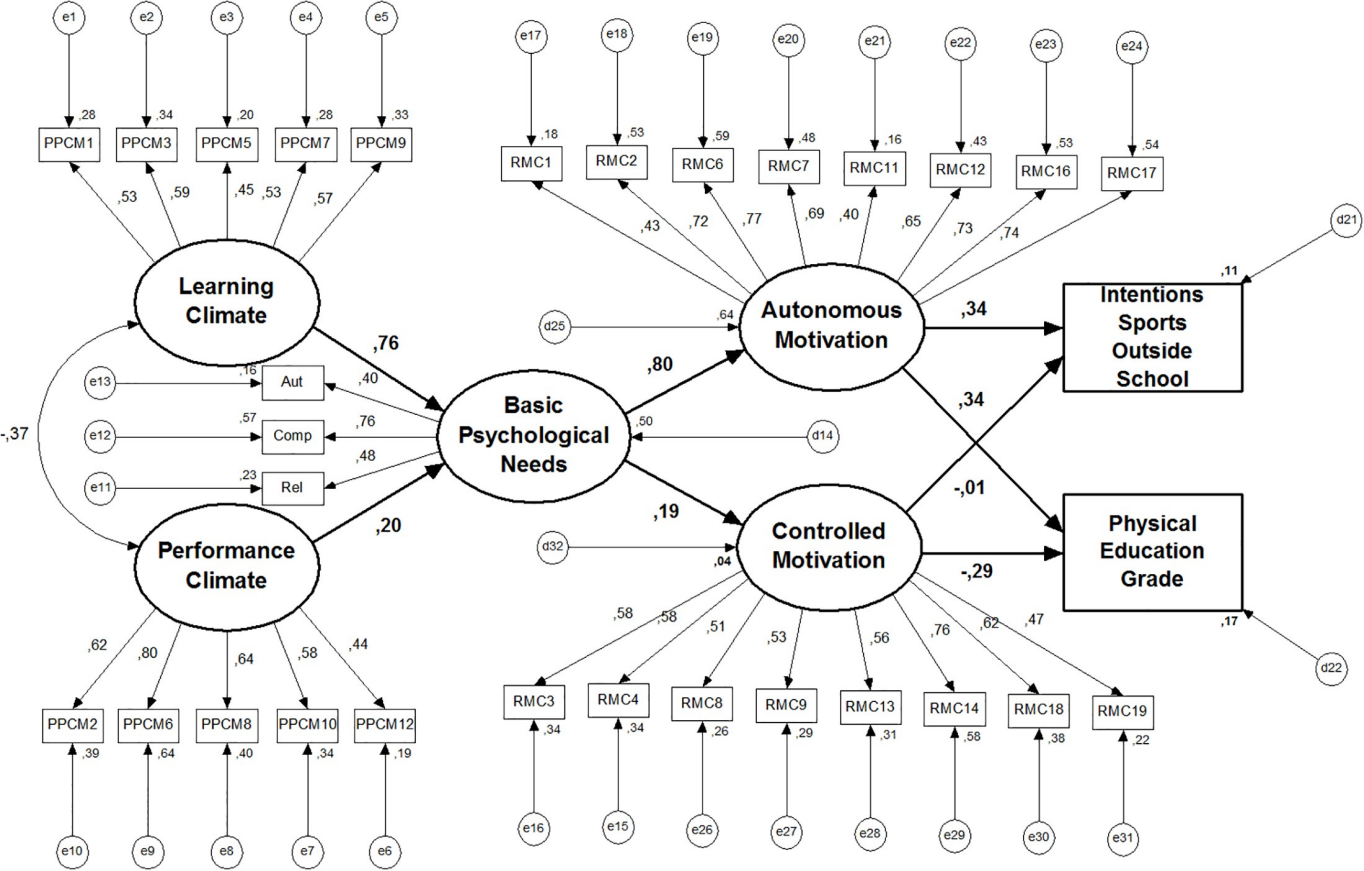
Model 1 (initially hypothesized) with standardized individual parameters.

**Fig 3:**
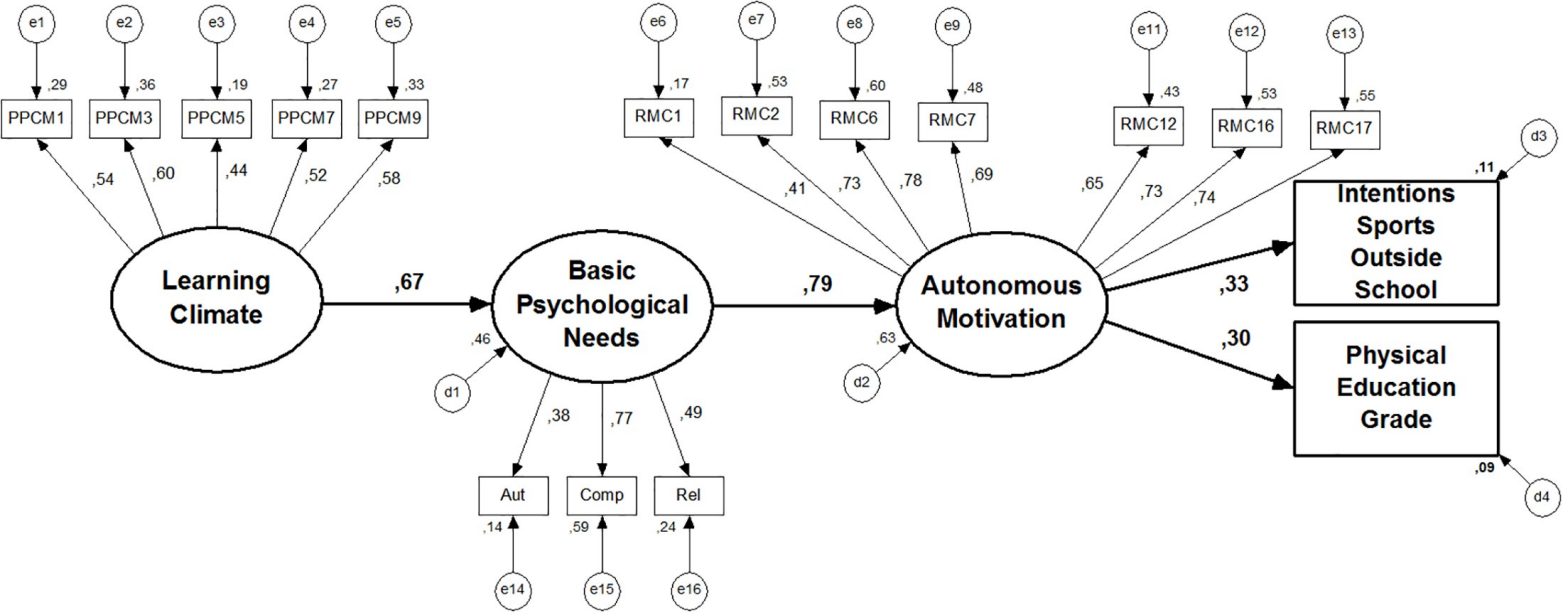
Model 2 (After elimination of the variables that cause instability in the model). Standardized individual parameters.

As it is seen in Fig 2, model 1 does not fit to data. The analysis of modification indices show us high residual values among all motivational climate items (oriented for performance) and all controlled motivation items suggesting instability in the model. In addition, there were also high residual values among some items of controlled motivation with the autonomous motivation, reinforcing model’s instability. Therefore, we decided to eliminate these two variables in the model (model 2—Fig 3). We also decide delete the item 11 (“…it’s exciting”) because of high residual values with item 1 (“… it’s fun”), both of intrinsic regulation, which seem to indicate something in common (similar semantic value). The elimination of item 11 led to model’s improvement.

**Fig 4:**
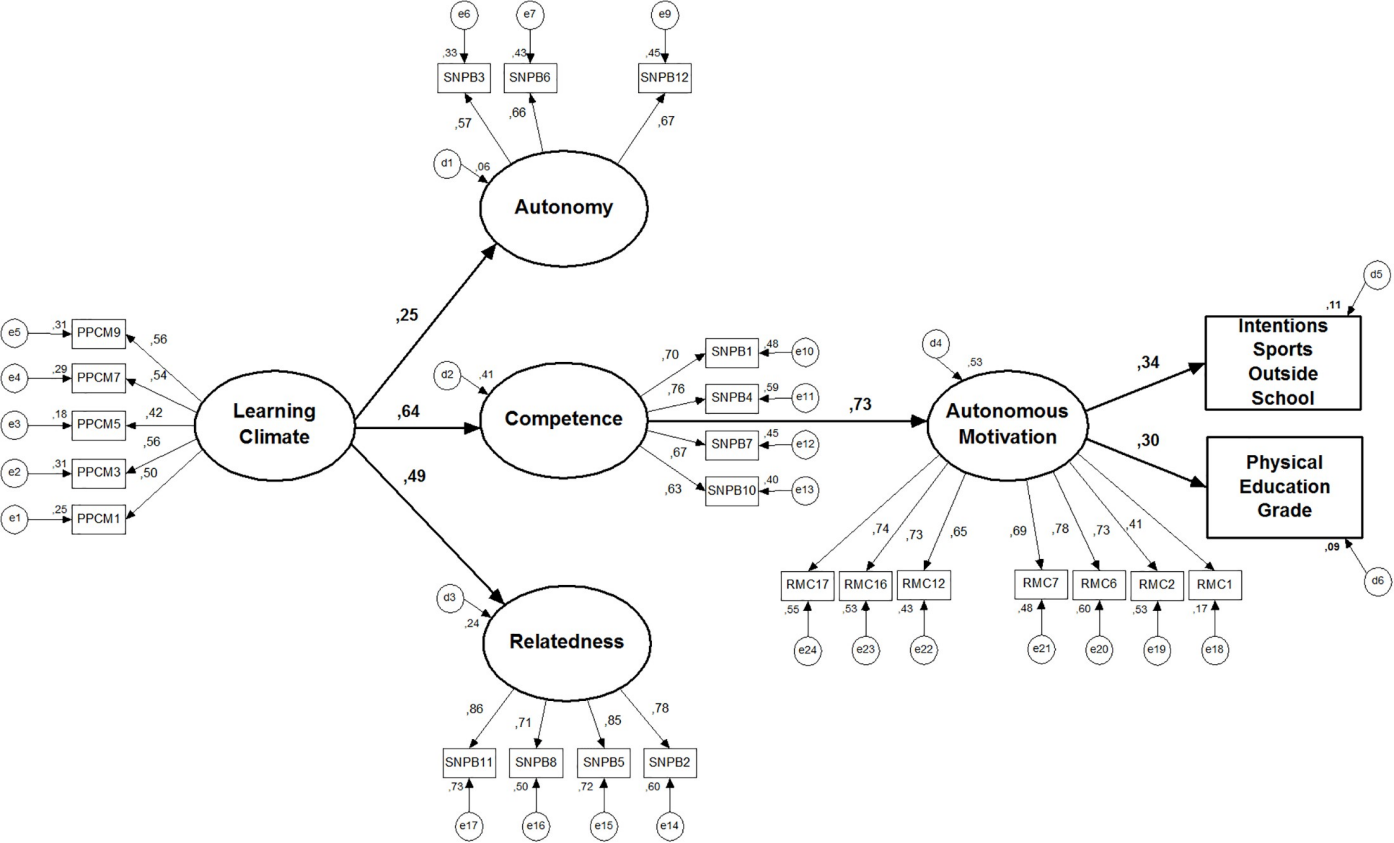
Model 3 (with the basic psychological needs analysed separately). Standardized individual parameters.

Regarding Fig 3, model 2 had acceptable fit to the data, considering the cut-off values proposed by Marsh et al. [42]. However, the analysis of indirect effects suggests a strong association of BPN, through autonomous motivation, to PE grade and intentions to practice sport in future. Furthermore, the need for "competence" is the strongest of the BPN (.77) and seems to have a central importance in this model. Therefore, we have decided to test our model with the three basic psychological needs separately (model 3—Fig 4). But, in doing so, some problems emerged (high residual values) between the item 9 (“autonomy”) and all the “competence” items. So, we decided to eliminate this item, a decision supported by the measurement model validation study of BPN in PE [36,37].

Despite the significant prediction of student´s perception of a learning motivational climate on all basic psychological needs, only the competence satisfaction had a significant direct effect on autonomous forms of motivation (β = .73). In turn, a significant positive prediction was found in students’ PE grade (final grade) (β = .30) and intentions for sport practice (β = .34). Competence presented a positive and significant indirect effect on students’ PE grade (β = .22) (through autonomous motivation), and their intention for future leisure-time sports participation (β = .24). In total, the model explains about 16% of the variance on PE grades and about 20% of the variance on intentions to practice sports in the future.

Results from the multi-group analysis (Table 4) showed that the structural model was invariant (p ≥ .05; ΔCFI ≤ .01). In other words, the model is equivalent across female and male students, thereby demonstrating that this model can be replicated in these samples.

**Table 4:** Goodness-of-fit-indices of structural invariance between genders.

Model 2 (Fig 3) M-F	χ^2^	df	Δχ^2^	Δdf	p	CFI	ΔCFI
Model 1	505.9	234	-	-	-	.908	-
Model 2	528.9	248	23	14	.06	.905	.003
Model 3	529.9	250	24	16	.08	.905	.003
Model 4	534.6	251	28.7	17	.04	.904	.004
Model 5	539.8	253	33.9	19	.02	.903	.005
Model 6	620.4	270	114.5	36	.01	.881	.027
Model 3 (Fig 4) M-F	χ^2^	df	Δχ^2^	Δdf	p	CFI	ΔCFI
Model 1	1121.1	542	-	-	-	.900	.000
Model 2	1140.9	562	19.8	20	.467	.900	.000
Model 3	1146.6	566	25.5	24	.374	.900	.000
Model 4	1152.2	567	31.1	25	.184	.899	.001
Model 5	1170.6	571	49.5	29	.010	.896	.004
Model 6	1267.2	596	146.1	54	< .001	874	.026

Legend: M = male; F = female; χ^2^ = chi-square; df = degrees of freedom; Δχ^2^ = differences in chi-square value; Δdf = differences in degrees of freedom; p = significance level; CFI = Comparative Fit Index; ΔCFI = differences in CFI value. Model 1: unconstrained model; Model 2: measurement weights; Model 3: structural weights; Model 4: structural covariances; Model 5: structural residuals; Model 6: measurement residuals

## Discussion

Considering AGT and SDT tenets, results seem to corroborate their theoretical frameworks. Some authors [10,11,12, 13] have already mentioned that individuals’ motivation is not directly predicted by social factors, but mediated by the satisfaction of three "*innate psychological nutriments that are essential for ongoing psychological growth*, *integrity and well-being*" ([14], p.229). These BPN will determine how someone regulates his own behaviour between a less or a more self-determined form (i.e., controlled vs. autonomous motivation). More self-determined behaviours influenced by BPNs’, are deemed to endorse and increase volitional participation in different domains of physical activity settings.

Thus, Deci and Ryan ([14], p.269) consider that there is a general convergence between AGT and SDT, since both theories suggest that "*environments that are less evaluative and more supportive of the intrinsic desire to learn provide the basis for enhanced achievement*". Therefore, the learning motivational climate promotes adaptive motivational patterns and is associated with increased psychological well-being and persistence in several behaviours [16, 46, 47, 48]. In short, a social context that supports autonomy (i.e., offering choices, supports the individual will and minimizes pressure and control) favours the satisfaction of BPN and consequently self-determined behaviour [10,11]. This is very important on different physical activity settings since "*intrinsic motivation may be among the most important factors in maintaining exercise over time"* ([12], p. 5).

In the face of theoretical explanation and support, empirical studies seem to support these results [49], presenting clarity in the association between AGT and SDT constructs in one comprehensive model. Furthermore, if we consider the results of several studies conducted in the physical activity domain (i.e., sport, exercise and PE), it is safe to say how the variables under analysis impact intention [20,24,25,26,27,28] and performance/behaviour [50,51,52]. Thus, if we consider PE grades as a positive performance / behaviour consequence, then our results are consistent with the available literature [53,54,55].

With regard to structural invariance between genders, the best practices recommended by several authors [40] regarding the re-specification of the model were followed. Guidelines recommend that when the hypothesised model does not fit the data, the re-specified final model should be tested in another sample using the same population to prove its validity and reliability. Thus, the final model that resulted from the analysis performed through the modification indices, was tested using another sample from the same population (i.e., between gender). The final model fit the data [40,41,42] and displayed gender invariance according to several recommendations, given that all of the criterion adopted in the methodology were achieved [40,43]. These results demonstrate that the theoretical constructs that underlie the structural model are perceived in the same way between male and female students, and that the causal relationships hypothesised in the model can be interpreted in the same way and with the equivalent predictive effect for both genders.

Considering our initial hypotheses, results support the empirical link between both theories under analysis. However, the proposed associations were only partially confirmed, since not all relations among variables were considered significant. However, the results allowed the following conclusions:

1. The motivational climate in PE classes, endorsed by the PE teacher, seems to have a significant impact on the satisfaction of BPN. The perception of a learning motivational climate (a context that places the emphasis on commitment, effort, cooperation and personal development) is a positive predictor of autonomy (the students are more able to regulate their own actions during the classes), competence (the students feel more effective in carrying out tasks / school activities) and relatedness relationship (the students feel more connected with peers);
2. BPN satisfaction seems to have a significant impact on how students regulate their behavior. Meeting BPN (particularly competence) is a positive predictor of autonomous motivation, including identified regulation (by which students feel more identified with the tasks/school activities, enhancing its benefits) and intrinsic motivation (the students derive great pleasure from school activities and have fun while doing it);
3. The way individuals regulate their motivation has a significant impact on their intentions in leisure-time physical activities and PE grades.

Therefore, it is essential to endorse in learning/teaching processes in PE classes, based on the current literature, present study findings, and authors’ professional experience. These guidelines stem with the notion of promoting an appropriate motivational climate in class, forecasting students’ BPN satisfaction and self-determined motivation:

1. In order to encourage a learning motivational climate, teachers can focus activities in the action itself and not on the result, so that students care more for the personal development of their motor skills / abilities. To do this, teachers should focus more on effort and less on results itself. Furthermore, cooperation and mutual aid between pairs (the task interdependence) should be emphasized, decreasing thereby the almost innate tendency of students to demonstrate their skills to others;
2. Teachers should increase the choice option in their students when facing tasks for developing autonomy. Pair work and small groups facilitate this process. Teachers must likewise explain to the students about the tasks to be undertaken, giving them the opportunity to choose the best way of performing;
3. Teachers should promote a learning climate based on observable references (demonstration of the task with or without the help of a volunteer student), as well as on indicators of learning evolution to develop competence;
4. Teachers should form small groups (considering student’s level of expertise), thus creating social bonds and encouraging cooperation among peers, in order to develop relatedness;
5. Regarding the development of more autonomous conducts, teachers should promote intrinsic motivation in PE classes, individualizing and adapting the teaching style to the characteristics and level of the students’ performance, as well as encourage them to actively participate in the decision-making process. Specifically, teachers should whenever possible address the students in a rational and logic explanation of the PE importance, facilitating the development of the identified regulation. The development of active lifestyles and the promotion of necessary motor skills for the acquisition of specific technical skills for a particular sport are results of autonomously forms of motivation.

In short, PE classes could play a key role in the fight against high rates of physical inactivity and sedentary lifestyle especially among Portuguese children and adolescents [56]. The results obtained from the hypothesized model emphasize the importance of three variables: learning motivational climate, satisfaction of basic psychological needs (especially competence), as well as self-determined motivation. As such, teachers should plan and develop their professional activity with the notion that a learning motivational climate induced in classes can influence effort, persistence, cognition, emotions and behaviour of students [28,48,53,55]. In addition, PE teachers should be aware of the importance of promoting competence among children, regardless of their skill level [30]. Nevertheless, they should be aware of promoting self-determined motivation, as a way to enhance the students intentions to be physically active [24,28,53].

Although the present study contributes on new insight on how motivational determinants predict intention to practice sports in future and PE grades, it has some limitations. All variables were assessed at one moment (cross-sectional design). Therefore, we cannot draw causality associations. Longitudinal and/or experimental studies are needed to further examine the effects of the analysed variables.

In order to increase knowledge on the effect of BPN in PE context, we suggest future studies considering the role of needs frustration on behavioural outcomes. Past studies [53] have shown that BPN frustration leads to negative outcomes, and we speculate that the frustration of autonomy, competence and relatedness could lead to decrease in student’s intention to participate actively in sports activities and lower grades in PE classes.

Lastly, forthcoming studies are encouraged to analyse the proposed model across other variables (e.g., age or academic level) to measure invariance.
